# Liquid–Liquid Phase Separation (LLPS) in Tumor Biology: Special Focus on the Process of Transcription

**DOI:** 10.3390/ijms27156766

**Published:** 2026-07-28

**Authors:** Wei Chen, Zihan Yang, Qiaodan Zhou, Bin Cheng

**Affiliations:** 1 Department of Gastroenterology and Hepatology, Tongji Hospital, Tongji Medical College, Huazhong University of Science and Technology, Wuhan 430030, China; m202476831@hust.edu.cn; 2 College of Basic Medical Sciences, Hubei University of Chinese Medicine, Wuhan 430065, China; 3376@hbucm.edu.cn; 3 Hubei Shizhen Laboratory, Wuhan 430065, China

**Keywords:** liquid–liquid phase separation, tumor, biomolecular condensate, transcription

## Abstract

Liquid–liquid phase separation (LLPS) has attracted considerable attention in cell biology as a potentially widespread organizing principle in the cellular environment. LLPS involving proteins and nucleic acids participates in a wide range of cellular processes, including regulation of genome activity, modulation of enzymatic activity, and control of subcellular compartmentalization. Emerging evidence supports the idea that aberrant LLPS behavior is associated with various diseases, including cancer and infectious diseases. These findings suggest that LLPS provides a new framework for understanding complex regulatory phenomena in cells. In this review, we provide a comprehensive overview of LLPS, focusing on the biophysical mechanisms underlying condensate formation, the molecular composition of biomolecular condensates, and current experimental approaches used to study this process. In particular, we highlight the role of LLPS in aberrant transcriptional regulation, with a specific focus on its regulatory functions and underlying molecular mechanisms in tumor cells. Collectively, this review provides an updated perspective on the functional and mechanistic roles of LLPS in physiological and pathological contexts, particularly in tumor biology.

## 1. Introduction

Since *Brangwynne CP* and his coworkers’ first analysis of liquid droplets in Caenorhabditis elegans in 2009, liquid–liquid phase separation (LLPS) has been widely recognized as a new principle for explaining cellular organization and regulation [1]. Important biological processes, such as the formation of membrane-less organelles (MLOs), the organization of the cytoplasm and nucleoplasm, chromatin architecture, mechanisms of gene regulation, and tumorigenesis, are increasingly being understood through the framework of LLPS. In 2011, T. Pederson proposed that membrane-free cellular structures, including the nucleolus, are maintained through weak interactions among their components [2]. Many of these components are highly flexible and lack stable three-dimensional structures, enabling them to form transient multivalent weak interactions and provide a flexible intracellular framework. In 2012, Rosen’s group used the Nck–N-WASP signaling system as a model and demonstrated that multivalent interactions between tandem repeat domains can drive liquid droplet formation, thereby elucidating the assembly mechanism of cellular signaling puncta [3]. Also in 2012, McKnight’s team showed that the intrinsically disordered regions (IDRs) of RNA-binding proteins, including FUS and TDP-43, mediate the formation of RNA-containing liquid droplets through weak intermolecular interactions, thereby establishing a mechanistic link between ribonucleoprotein granules and pathological protein aggregation in neurodegenerative diseases [4]. Together, these landmark studies laid the theoretical foundation for subsequent research on LLPS in biological systems.

Despite their high internal dynamics, most condensates maintain relatively stable overall size and morphology while continuously exchanging constituent molecules with the surrounding cytoplasmic or nucleoplasmic environment on the timescale of seconds [5,6]. This property can therefore be used to detect biomolecular condensates experimentally. To visualize protein phase separation, researchers commonly use fluorescent protein tags or other fluorescence-labeling strategies to monitor the localization, morphology, and dynamics of condensates in both living cells and reconstituted systems [7]. However, in many studies, fluorescent protein tags observed in vivo or in vitro have been used only as qualitative or indirect evidence. Among these observational methods, fluorescence recovery after photobleaching (FRAP) is widely regarded as the “gold standard” for LLPS analysis, although several important caveats must be considered when interpreting the results [8].

The process of LLPS is regulated by multiple factors that influence protein multivalent interactions, including molecular alterations, changes in the activity of phase-separation regulators, and variations in physicochemical conditions [9]. Post-translational modifications (PTMs), such as phosphorylation, methylation, ubiquitination, and oxidation, can alter protein charge and the strength of intermolecular interactions [10]. Thus, as a general mechanism for integrating diverse signals, PTMs can regulate LLPS and phase transitions, thereby tuning the properties and functions of biomolecular condensates [11]. Specifically, PTMs may either promote or inhibit LLPS under both physiological and pathological conditions by modulating protein charge and interaction strength [10].

In recent years, significant progress has been made in understanding the molecular mechanisms underlying cancer initiation and progression. Aberrant protein activities can drive cancer cells toward immortality or, in some contexts, restrain their progression. Phase separation provides a new framework for interpreting and understanding cancer phenotypes. In this review, we discuss the role of LLPS in various cancers, with a particular focus on the LLPS-mediated assembly of transcription factor complexes. For example, YAP/TAZ proteins recruit the Mediator complex to promote CDK9-dependent transcriptional activity at target loci [12].

To provide a systematic overview of LLPS, this review summarizes its biophysical features, biological functions, and detection methods. We hope to demonstrate that phase separation provides a useful framework for understanding transcriptional regulation and ultimately for developing new therapeutic strategies against human cancer.

## 2. The Components and the Biophysical Principles of LLPS

The mechanisms underlying the occurrence, organization, and material properties of LLPS condensates have long remained elusive. However, with the development of technologies that enable the direct detection of phase transitions, significant progress has been made in understanding the principles of LLPS both in vivo and in vitro. LLPS is a process in which biomacromolecules separate from the homogeneous intracellular milieu to form distinct coexisting liquid phases with different molecular compositions and material properties [13]. These liquid phases often exchange components dynamically with the surrounding intracellular fluid. During LLPS, the valency of interacting components and the number of interaction domains play a key role [9]. Thermodynamic and polymer-physics frameworks have provided important conceptual foundations for understanding LLPS. In particular, the Flory–Huggins theory offers a useful description of the balance between entropic mixing and enthalpic interactions that determines whether macromolecules remain mixed or demix into coexisting phases [14]. Related developments in polymer theory have further informed current models of multivalent biomolecular condensation. In the biological context, these concepts help explain how concentration, interaction valency, solvent conditions, and sequence features collectively regulate condensate formation [15,16]. Under normal circumstances, biomacromolecules in a solution interact with one another and with solvent molecules in a manner that minimizes the free energy of the system. When the solute concentration of the solution exceeds a critical threshold, the system undergoes an abrupt state change. The solution undergoes phase separation, resulting in a dilute solution phase and a condensed, biomacromolecule-rich phase [17].

In addition to liquid–liquid phase separation, other types of phase transition, such as gelation, also occur in biological systems. Gelation is a kind of connectivity transition that involves the formation of a system-spanning network of interactions [18]. For example, extensive hypomethylation induces FUS condensation into stable intermolecular β-sheet-rich hydrogels, a process that disrupts RNP granule function and impairs new protein synthesis at neuronal terminals [19]. Importantly, phase separation and gelation often occur concurrently. In such a case, the molecules which own a structure characteristic of phase separation involve in both the LLPS system and the gelation system simultaneously [9].

Although the molecular architectures and precise domains involved in phase separation remain incompletely defined, recent studies have identified several common features of proteins that undergo phase separation, as exemplified by T-cell receptor (TCR) signaling clusters [20], gel-like assemblies in the postsynaptic density [21], and Numb and Pon condensates in Drosophila [22]. These proteins serve different functions in diverse biological processes, but they all exhibit multivalency. Studies have shown that protein multivalency strongly influences the formation of biomolecular condensates through LLPS [23]. The multivalency of these proteins often arises from repetitive amino acid motifs within intrinsically disordered regions (IDRs) [24], which contribute to reversible assembly and the formation of membrane-less organelles (MLOs) or stress granules [25,26]. Proteins containing substantial IDRs are generally referred to as intrinsically disordered proteins (IDPs) [27]. Because IDRs are enriched in a limited set of specific amino acid residues, many IDPs lack stable tertiary structures. This polar amino acid composition typically includes glycine, polar residues (e.g., glutamine and serine), and aromatic residues (e.g., tyrosine and phenylalanine) [13,28]. Whether a protein is classified as an IDP can often be inferred from its amino acid composition. Deciphering the distinct protein-intrinsic codes that govern the formation of various condensates remains a major challenge in the field [9].

The biomacromolecular phase separation is not limited to proteins. RNA is an important participant in the formation of RNA/protein droplets at physiological conditions [16]. Multiple studies have shown that various types of RNA are both necessary and, in some contexts, sufficient for the formation of ribonucleoprotein (RNP) droplets [3,25,29,30]. Recent evidence indicates that elevated nuclear RNA concentration prevents FUS from undergoing liquid–liquid phase separation in mammalian cells [31]. In paraspeckles, overexpressed long non-coding RNAs (lncRNAs) can interact directly or indirectly with RNA-binding proteins (RBPs) to regulate cellular functions by altering LLPS behavior [32]. Furthermore, certain mRNAs can participate in homologous or heterologous interactions within cells, thereby promoting the formation of droplet-like structures [33]. These results suggested that RNA can exert distinct effects on cellular processes depending on its local abundance.

**Figure 1:**
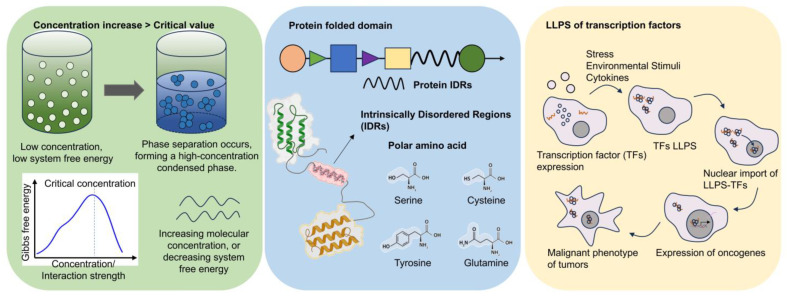
Schematic illustration of LLPS at multiple biological scales. (**Left**): the relationship between system free energy and solute concentration during LLPS from a physicochemical perspective. (**Middle**): the contribution of key polar amino acids within intrinsically disordered regions (IDRs) to LLPS at the protein level. (**Right**): the role of LLPS in tumor cells, including the promotion of transcription factor nuclear import and activation of downstream signaling pathways.

RBP–RNA interactions are key to maintaining the liquid-like state of condensates, thereby enabling their dynamic reorganization and functional plasticity. For instance, in early embryos, RNA reduces the viscosity of LAF-1 droplets and enhances their molecular dynamics [30]. Zhang et al. demonstrated that Whi3 targeting of mRNA under physiological conditions modulates key physical properties of phase-separated droplets in vitro, including viscosity, fusion dynamics, and the rate of component exchange with the solvent [34]. The mechanisms by which RNA features influence biophysical properties and drive the formation of RNA-dependent phase-separated droplets are now being extensively investigated (Figure 1) (Table 1).

## 3. How to Detect LLPS

While the biological significance of LLPS of various biomacromolecules is currently being extensively investigated, the approach to unravel the underlying biophysical mechanisms of the phase separation droplets is still in its infancy. Assessing the assembly state of biomacromolecules is challenging, and many factors can influence the formation of liquid–liquid phase-separated condensates. Changes in the concentration of target biomolecules, ionic strength of the buffer, system temperature, and intra- or intermolecular interactions can all quantitatively regulate the propensity of a polymer to demix [48]. Some slight environmental disturbances could promote or disrupt the condensation of biological macromolecules in a short period of time. Therefore, we need more precise and reproducible assays to detect phase-separated condensate-forming processes in vivo or in vitro with small environmental changes.

Early works on polymer physics laid the groundwork for the subject of phase separation in biology, which was subsequently advanced by structural and biophysical techniques [49]. To demonstrate how LLPS systems regulate cellular condensates, a number of elegant in vitro experiments have been designed and conducted [13,50,51]. A range of biophysical techniques—including computational simulations (molecular dynamics), magnetic resonance (NMR, EPR), scattering methods (DLS, SAXS), and single-molecule fluorescence spectroscopy—have become essential for deciphering the molecular mechanisms underlying the behavior of biomacromolecules in phase-separated states [52,53]. Among these techniques, NMR has emerged as a leading method, offering insights into the structure, dynamics, and interactions of proteins at a residue-specific level in both dispersed and condensed phases, an area that has been extensively reviewed [54]. Despite numerous biochemical studies revealing the physical properties of macromolecules undergoing LLPS, the extent to which LLPS occurs within the crowded cellular environment remains unclear.

Fluorescence recovery after photobleaching (FRAP) is frequently used to examine the diffusivity of molecules inside and outside droplets, as well as to assess their ability to undergo LLPS in vitro [55]. According to the FRAP principle, photobleached molecules diffuse out of the bleached region and are replaced by new fluorescent molecules, thereby restoring the fluorescence signal [56]. FRAP experiments can provide evidence supporting LLPS by measuring molecular mobility and the dynamics of molecular interactions. FRAP experiments do not by themselves establish the presence of LLPS; rather, they are commonly used to assess the dynamic exchange and liquid-like behavior of condensates by measuring fluorescence recovery after photobleaching. Therefore, FRAP should be interpreted together with additional evidence, such as condensate morphology, reversibility, concentration dependence, and orthogonal perturbation assays. The fluorescence recovery time reflects various fundamental kinetic properties of molecules within an LLPS system. The mobile fraction and recovery rate of fluorescent molecules can be readily determined by FRAP, both of which are key parameters in the study of protein movement [57]. As a result, FRAP is often regarded as a “gold standard” method for assessing LLPS-associated molecular dynamics.

Although FRAP is widely used in LLPS research, several limitations should be considered when interpreting the results. 1. Fluorescence recovery can occur not only from freely diffusing molecules in solution but also from proteins engaged in stable, high-affinity interactions [58,59]. 2. The kinetics of fluorescence recovery are influenced by complex interactions among multiple parameters, including, but not limited to, the diffusion coefficient and concentration of the molecule under study, its binding rate to interaction partners and the diffusion properties of those partners, the number and affinity of binding sites, and technical parameters of the microscope and detection system. Variation in any of these parameters can significantly affect the rate of fluorescence recovery within the bleached region [60]. 3. Fluorescent tagging may perturb the native properties of the protein being studied. It was discovered that GFP made the best confocal FRAP probe. In most cases, the target protein is fused to GFP at either the N terminus or C terminus to enable FRAP analysis. Such tagging may alter the physicochemical properties, localization, or interactions of the protein. Other fluorescent dyes, including fluorescein isothiocyanate, are also prone to photobleaching. However, fluorescent dyes must be coupled to a protein by a cell permeant antibody or lectin since they can generate harmful free radicals that can affect protein behavior and cell health [57]. Therefore, fluorescent dyes also have important limitations in FRAP-based analyses.

The detection of LLPS formation by the properties of viscosity is also one of the common techniques used today. Another crucial measure to reflect the characteristics of the LLPS system is viscosity. Shana Elbaum-Garfinkle et al. adapted a microrheology technique to precisely measure the viscoelasticity of micrometer-sized LAF-1 droplets, revealing predominantly viscous properties that are highly tunable by salt and RNA concentrations [30]. Nicole Taylor et al. employed a microfluidic platform to confine protein droplets within a uniform macroscopic phase, thereby simplifying viscosity assessment through either passive microrheology or active two-phase flow analysis. Furthermore, this system enables both active and passive microrheological measurements to be performed within the same device, providing a critical methodological foundation for investigating how ATP-dependent biological activities regulate ribonucleoprotein (RNP) droplets [61].

Quantitative assessments of absolute protein abundance are now possible, with or without fluorescent labeling, owing to advances in light microscopy and spectroscopy [62]. Force spectroscopy techniques also allow for the assessment of LLPS droplet mechanical properties, such as stiffness and elastic modulus [63,64]. Atomic force microscopy (AFM), for instance, may provide a variety of capabilities, from surface imaging to the detection of contact forces, and it can also supply quantitative characteristics that can characterize alterations that are typical of many illnesses, including cancer [63].

**Figure 2:**
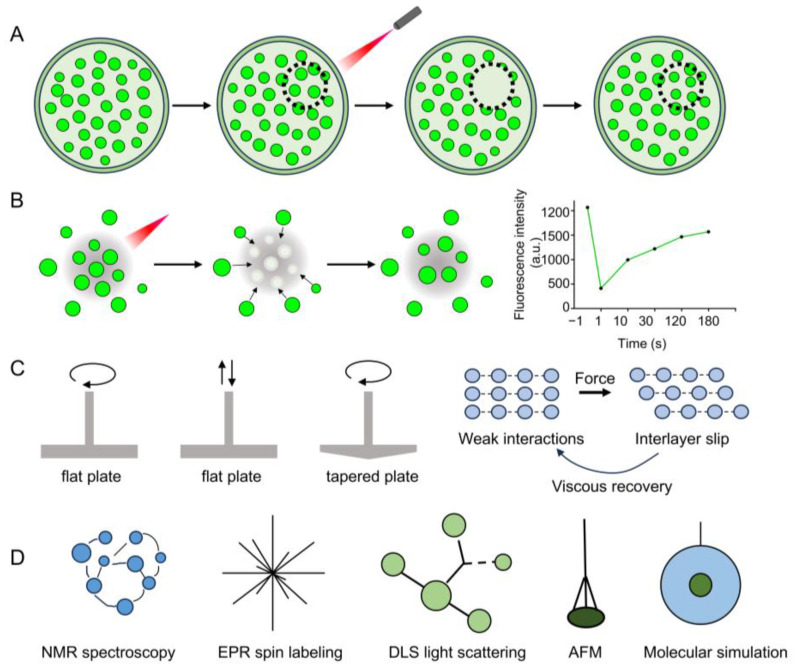
Techniques for detecting phase separation. (**A**) Fluorescence recovery after photobleaching (FRAP), a widely used method for analyzing protein dynamics both in vitro and in cells. (**B**) Schematic illustration of the principle underlying FRAP. (**C**) Representative methodologies for assessing droplet viscosity and related material properties. (**D**) Other commonly used techniques and approaches for detecting phase separation.

Recently, the discovery of an alternate compartmentalization mechanism for the herpesvirus has been facilitated by single molecule tracking experiments [65]. These results suggest that single-particle tracking methods may be successfully used in other systems to study the influence of hypothesized phases on molecular behavior [8]. Although endogenous states and physiological abundance can now be measured using numerous approaches, techniques for examining the material and mechanical properties of condensates still require further development (Figure 2).

## 4. The Function of LLPS in Cancer

**Figure 3:**
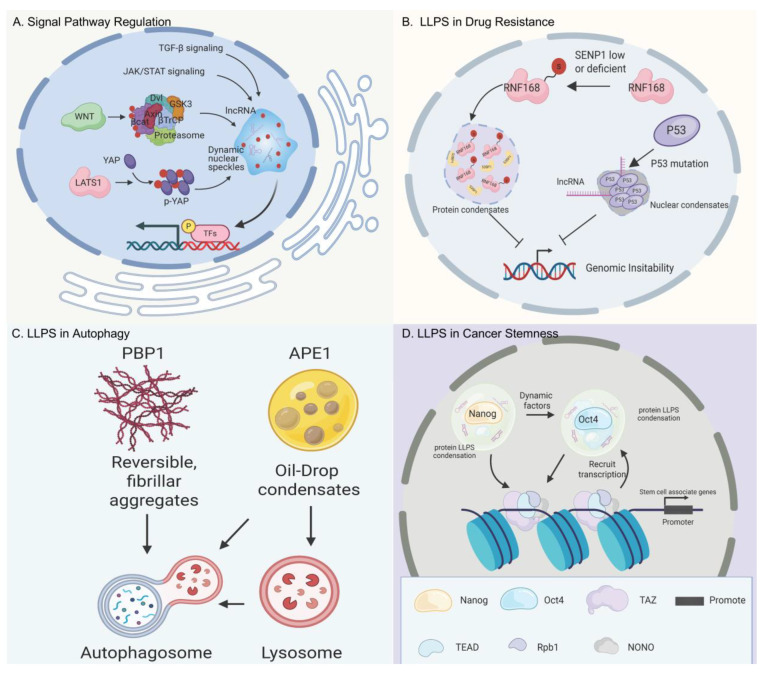
Functions of LLPS in cancer. (**A**) Phase separation in tumor cells facilitates transcription factor activity and thereby modulates tumor progression. (**B**) In tumor cells, p53 can undergo lncRNA-scaffolded phase separation, while RNF168 and 53BP1 also participate in phase separation processes associated with drug resistance. (**C**) Autophagy in tumor cells can be modulated by phase separation of proteins such as APE1 and PBP1, which facilitates lysosome-mediated autophagy initiation. (**D**) NANOG, OCT4, and TAZ are key regulators of phase separation in cancer stem cells. Phase-separated droplets formed by NANOG and OCT4 facilitate the enrichment of YAP signaling-related proteins at transcription initiation sites, thereby driving high expression of cancer stem cell-associated genes.

In recent years, significant progress has been made in our understanding of the molecular mechanisms of cancer. In contrast to the extensive research on cancer signaling pathways, comprehensive causal investigations of protein phase separation and biomolecular condensates in cancer cells remain at a very early stage. Accumulating evidence suggests that various cellular condensates form through liquid–liquid phase separation, thereby facilitating complex multiphase organization within cells. It is anticipated that additional biological functions mediated by these biomolecular condensates will continue to be discovered in the future (Figure 3).

### 4.1. Regulatory Signaling Pathway

Phase separation and condensate formation have recently emerged as important regulators of cellular signal transduction. Liquid–liquid phase separation of proteins generates distinct physical and biochemical compartments, thereby facilitating signaling. Numerous studies have shown that key proteins involved in intracellular signal transduction can undergo LLPS, which may either activate or inhibit signaling pathways and thereby contribute to the precise regulation of tumor cell behavior. To further explore the impact of phase separation of core signaling pathway proteins on tumor cell progression, we summarize the major signaling pathways involved (Table 2).

NF-κB is a well-known family of inflammation-related transcription factors. Upon stimulation by cytokines, stress, or oncogenic signals, IκB kinase (IKK) phosphorylates IκB, leading to its degradation and the initiation of downstream gene transcription involved in diverse biological processes, including inflammation, proliferation, apoptosis, immunity, and metabolism [66]. Liu et al. revealed that in hepatitis B virus-associated hepatocellular carcinoma, PrPC recruits downstream proteins through LLPS to activate the NF-κB pathway and accelerate HCC progression. They further showed that the α3 helix of PrPC is essential for NF-κB activation during LLPS, ultimately increasing IL-8 expression and promoting tumor development [67]. Moreover, in oral squamous cell carcinoma, DDX41 undergoes LLPS with STING to form biomolecular condensates, which promotes PD-L1 upregulation via the STING-TBK1-NF-κB signaling pathway; acting as a cytoplasmic DNA sensor, DDX41 maintains the activation of this signaling cascade and thereby mediates immune evasion in oral squamous cell carcinoma [68].

To interact with Mediators and target specific genes, Zamudio et al. demonstrated that core components of the WNT, TGF-β and JAK/STAT signaling pathways rely on their intrinsically disordered regions (IDRs) to achieve this biological function [69]. Upon signaling stimulation, these signaling molecules are recruited into biomolecular condensates together with coactivators at super-enhancer-controlled genes in a cell-type-specific fashion [69]. Moreover, disease-associated mutants of the non-receptor protein tyrosine phosphatase SHP2 can recruit and activate wild-type SHP2 within LLPS condensates, thereby promoting activation of the MAPK signaling pathway [70]. Li et al. uncovered a novel regulatory role for long noncoding RNAs (lncRNAs), showing that they facilitate the LLPS of the signaling kinase LATS1 and mediate YAP phosphorylation, thereby regulating the Hippo signaling pathway and promoting breast cancer progression [71]. Su et al. found that downstream signaling proteins spontaneously segregate into liquid-like clusters in response to T cell receptor (TCR) phosphorylation, thereby increasing signaling output both in vitro and in human Jurkat T cells [20]. Manipulating the phase behavior of such oncogenic proteins through specific methods could be a promising approach for treatment.

**Table 2:** Influence of phase separation of core signaling proteins on tumor progression.

Tumor Type	Core Protein	Key Findings	References
**NF-κB signaling**			
Lung squamous cell carcinoma (LUSC)	L1-ORF1p	1. L1-ORF1p from pathogenic LINE-1 forms condensates. 2. It scaffolds cGAS-HMGN2 complexes to activate cGAS-STING. 3. This axis drives immunosuppression in lung squamous cell carcinoma.	[72]
Oral squamous cell carcinoma (OSCC)	DDX41 STING	1. DDX41 acts as a cytosolic DNA sensor in OSCC. 2. DDX41 sustains the STING-TBK1-NF-κB pathway. 3. This mechanism facilitates PD-L1-driven tumor immune escape in OSCC.	[68]
Liver cancer	Cellular prion protein (PrPC)	1. PrPC is upregulated in HBV-positive HCC, induced by HBx-CREB1 axis. 2. PrPC undergoes LLPS to recruit downstream proteins and activate NF-κB, promoting HCC progression. 3. PrPC α3 helix and C-terminal disulfide bonds are critical for its LLPS and NF-κB-mediated IL-8 elevation.	[67]
**Wnt/β-catenin signaling**			
Lung cancer	AFAP1-AS1	1. AFAP1-AS1 regulates RNA alternative splicing by modulating splicing factor phase separation. 2. This mechanism drives drug resistance in lung adenocarcinoma. 3. AFAP1-AS1 and its controlled splicing events are potential biomarkers and therapeutic targets.	[73]
Colorectal cancer	LEF1	1. LEF1 forms LLPS condensates with β-catenin upon Wnt activation in colorectal cancer cells. 2. LEF1 IDR domain is required for LLPS and downstream target gene transactivation with β-catenin. 3. LEF1 facilitates colorectal cancer cell proliferation and migration in an IDR-dependent manner.	[74]
Prostate cancer	DACT1	1. TGFβ-induced DACT1 forms cytoplasmic phase-separated condensates to inhibit Wnt signaling. 2. Intrinsically disordered domains of DACT1 are essential for condensate formation and Wnt repression. 3. DACT1 condensates sequester CK2 to block Wnt activation, and DACT1 drives breast and prostate cancer bone metastasis.	[75]
**RAS-MAPK signaling**			
	SHP2	1. Disease-related SHP2 mutants acquire LLPS ability to enhance its PTP enzyme activity. 2. SHP2 LLPS is driven by PTP domain electrostatic interactions and controlled by intramolecular conformation. 3. Mutant SHP2 recruits wild-type SHP2 into condensates to boost RAS-MAPK signaling. 4. SHP2 allosteric inhibitors suppress mutant LLPS; targeting LLPS is a potential therapeutic strategy.	[70]
Papillary thyroid carcinoma	CCDC6-RET	1. The CCDC6-RET fusion kinase undergoes intrinsic LLPS, which is coupled with its kinase activity. 2. RET fusion drives persistent activation of the Ras/MAPK pathway. 3. LLPS of RET fusion forms a ternary complex with GRB2 and SHC1 to sustain Ras/MAPK signaling.	[76]
**AMPK signaling**			
Breast cancer	FOXM FKH	1. FOXM1 forms LLPS condensates to sustain chromatin accessibility and promote breast cancer metastasis. 2. AMPK phosphorylation of FOXM1 IDR disrupts LLPS, represses oncogenic transcription and activates anti-tumor immunity. 3. Targeted peptide abolishes FOXM1 LLPS, inhibits tumor progression and enhances immunotherapy efficacy.	[77]
Liver cancer	YAP	1. Endogenous YAP forms LLPS condensates in mouse primary liver cancer. 2. YAP phase separation drives multiple liver tumor subtypes by suppressing AMPK signaling. 3. A TEAD1-derived peptide disrupts YAP condensates, activates AMPK and inhibits liver cancer progression.	[78]

### 4.2. Drug Resistance

A major challenge in treating various cancers is drug resistance. Drug-resistant cancer cells employ multifaceted molecular strategies to counteract therapeutic interventions under drug pressure [79]. Recent studies have shown that LLPS plays an important role in therapeutic responses in several cancers, including lung, colorectal, and breast cancer. Structural mutations in oncogenic proteins, as well as alterations in LLPS-associated intrinsically disordered regions (IDRs), can critically affect various cellular functions, including membraneless organelle formation, oncogene expression, and responses to pharmacological stress and other stimuli.

In lung cancer, LLPS promotes drug resistance by facilitating the formation of biomolecular condensates that sequester tumor suppressor proteins. For instance, the tumor suppressor p53 has been shown to undergo LLPS, generating condensate droplets that activate downstream effectors of the DNA damage response. However, p53 mutations can impair the assembly of these condensates, leading to compromised DNA repair and elevated genomic instability [80]. In colorectal cancer, Sentrin/SUMO-specific protease 1 (SENP1) decreases RNF168 SUMOylation in response to DNA damage and prevents RNF168 from undergoing LLPS to form nuclear condensates, thereby impairing DNA repair; depletion of SENP1 sensitizes cancer cells to chemotherapy [81]. In breast cancer, Huang et al. explored the roles of LLPS-correlated genes (LCGs) in the prediction of breast cancer prognosis. The results showed that the LCG-based risk-scores and clinical factor-based prognosis prediction tools had medium accuracy, which means that LLPS-correlated genes are powerful for constructing a prognosis prediction model [82]. Elucidating the role of liquid–liquid phase separation in cellular processes may reveal novel therapeutic avenues for counteracting drug resistance in cancer.

### 4.3. Autophagy

Autophagy is a fundamental cellular degradative pathway that occurs in all eukaryotic cells. In order to maintain cellular homeostasis and adapt to changing nutritional circumstances, it is utilized to recycle cytoplasm, eliminate unnecessary organelles, and repair damaged organelles. Macroautophagy is the most prevalent form of autophagy [83]. Among the process of autophagy, LLPS plays important roles in multiple steps of it. For example, the low-complexity region of PBP1 (polyA-binding protein 1) forms reversible fibrils through the LLPS, and facilitates self-assembly into structures essential for TORC1 inhibition in yeast. Yang et al. introduced mutations into the protein and weakened its phase separation in vitro. Their results showed that the capacity to inhibit TORC1 and induce autophagy was reduced [84]. Moreover, APE1 (aminopeptidase I), a selective autophagy cargo, undergoes LLPS to generate liquid-like droplets. The APE1-specific receptor ATG19, in conjunction with lipidated ATG8, is both necessary and sufficient for membrane-mediated sequestration of APE1 droplets. Thus, the fluid nature of autophagic cargo and the presence of specific receptors on its surface are critical determinants for the selective autophagy of biomolecular condensates [85]. The abnormal mutant of the structure in protein IDRs could lead to cell dysfunction. Accumulation of semi-liquid protein condensates is a critical process in the pathogenesis of protein aggregation diseases. Designing therapeutic approaches to reduce the buildup of abnormal protein aggregates will be made easier by learning more about the specification and control of the biophysical features of protein condensates [11].

### 4.4. Cancer Cell Stemness

Cancer stem cells (CSCs) may be the most relevant and elusive cancer cell population, as they represent a major source of treatment resistance and tumor progression. Aberrant expression of stemness genes, such as NANOG, OCT4, and SOX2, which encode transcription factors essential for maintaining the pluripotent embryonic stem cell phenotype, is associated with suppressed apoptosis, therapeutic resistance, and drug resistance. Emerging evidence indicates that various stemness-related transcription factors engage with the Mediator complex through the LLPS activity inherent in their activation domains, and that the ensuing condensate assembly plays a direct role in transcriptional activation [86]. Wei et al. showed that the paraspeckle protein NONO is required for LLPS and nuclear activation of TAZ, a key effector of the Hippo signaling pathway. Accordingly, depletion of NONO reduces interactions between TAZ with TEAD, Rpb1, and other enhancers. In glioblastoma, increased TAZ phase separation is associated with poor prognosis [87]. Research has demonstrated that the stemness transcription factor OCT4 promotes prostate carcinogenesis by facilitating the assembly of a multi-protein complex comprising Forkhead box protein A1 (FOXA1), androgen receptor (AR), and nuclear respiratory factor 1 (NRF1). The transcription factors’ cooperation through the transcriptional activation-underlying LLPS contributes to the aggressiveness and treatment resistance of cancer [88].

### 4.5. Chromatin Fusion and Epigenetic Change

Recurrent gene fusions involving IDR-containing and chromatin-binding proteins are an important feature of many cancers [89]. Gene fusions involving IDR-containing and chromatin-binding proteins are frequently observed across many malignancies. Growing evidence shows that changes to three-dimensional chromatin structure caused by the phase separation of chromatin-associated molecules might affect gene transcription [90,91,92]. As proposed by Gibson et al., phase separation may establish a condensed yet plastic chromatin ground state, from which diverse “excited” structural configurations can emerge through covalent modifications and regulatory inputs, thereby enabling functional plasticity of the genome [93]. These findings illustrate how LLPS can generate chromatin domains that are spatially proximal yet functionally segregated in vivo, suggesting that the intrinsic capacity of chromatin to undergo phase transitions may play a fundamental role in eukaryotic genome organization and regulation [93]. One example is the fusion between the IDR of EWS and the FLI protein in Ewing sarcoma. In Ewing sarcoma, the BRG1/BRM-associated factor (BAF) complex is recruited to tumor-specific enhancers by the EWS-FLI1 fusion protein, where it facilitates the activation of target genes. This recruitment represents a distinctive function of the EWS-FLI1 fusion and is mediated by specific tyrosine residues within the prion-like domain of EWSR1 that are essential for its phase-separation capability [94].

## 5. The LLPS Model in Transcriptional Control

The processes of transcription regulation involved in the fundamental cellular process have been thoroughly investigated, whereas those involved in its selectivity have received less attention. Super-enhancers (SEs) are clusters of transcriptional enhancers occupied by exceptionally high densities of transcriptional machinery and regulate target genes with particularly important roles in cell identity [95,96]. Recent studies have revealed that LLPS-mediated transcriptional condensates can assemble large numbers of transcription factors (TFs), cofactors, and RNA polymerase II (RNA Pol II) at SEs [86,97,98]. The transcribing enzyme RNA polymerase II may interact with other TFs and cofactors, shuttle between these LLPS condensates in a phosphorylation-dependent manner, and regulate the transcription of target genes.

### 5.1. The Super-Enhancers (SEs) and LLPS

Transcriptional dysregulation represents a cornerstone of cancer pathogenesis, driven by alterations in both coding sequences and noncoding regulatory elements. Super-enhancers (SEs), as large-scale transcriptional regulatory hubs, play a pivotal role in maintaining cancer cell identity and sustaining oncogenic gene expression programs, leading to a pronounced dependency of cancer cells on these regulatory structures [99]. SEs are defined as expansive genomic regions comprising densely clustered enhancer elements, which are extensively enriched with histone modifications and occupied by high densities of transcription factors and coactivators. These structures function as potent oncogenic drivers, substantially amplifying transcriptional output to sustain malignant phenotypes. In general, typical enhancers are relatively short, usually spanning 100–1000 bp of DNA, whereas SEs occupy extended genomic regions longer than 10 kb and can sometimes encompass epigenomic landscapes extending beyond 100 kb [96,100,101]. Conventional transcription factors alone cannot effectively engage with super-enhancers (SEs) to fully determine their functional state or regulate the transcriptional activity (activation or repression) of their target genes. Therefore, beyond direct DNA binding and transcription factor interactions, a complex network of protein–protein associations is established on SEs, which is modulated by the DNA-binding affinity, specificity, and allosteric conformations of transcription factors [102,103,104]. The structure of higher-order transcriptional regulatory complexes depends on the affinity-dependent ability of TFs to assemble multi-protein complexes, recruit cofactors, and remodel or modify chromatin landscapes [105].

Owing to the structural and functional properties of SEs, some TFs containing intrinsically disordered regions (IDRs) can promote the assembly of transcriptional components by driving LLPS into transcriptional condensates at SE sites [106]. During the beginning of the transcription process, the Mediators and RNA Pol II are colocalized in area-restricted stable clusters, which associate with SE sites and have properties as LLPS condensates in a transcription-dependent manner. With the help of the assembled stable TF-Mediator-Pol II clusters, organization of the genome in 3D and the SE function are closely interlinked. Recent studies have uncovered that the post-translational modifications of RNA polymerase II correlate with different stages of transcription [107]. Phosphorylation of Ser5 residues within the C-terminal domain of RNA polymerase II is associated with initiation condensates and plays an important role in LLPS formation. The Ser5-phosphorylated-RNAPII complex is associated with the nuclear phosphatidylinositol 4,5-bisphosphate (PIP2), and facilitates the RNAPII transcription [107]. Either the structure change of RNAPII or TFs can affect the transcription efficiency. Consequently, disrupting transcription factor cooperativity within LLPS condensates at super-enhancers may represent a viable therapeutic strategy in oncology.

In summary, an important emerging concept is that SEs function as privileged sites for condensate assembly, where enhancer activity, chromatin topology, and transcriptional output are tightly coupled. This perspective shifts the interpretation of SEs from static genomic features to dynamic regulatory hubs whose functions depend on multivalent molecular interactions and phase behavior.

### 5.2. The LLPS in Transcription Factors

The process of transcription initiation in eukaryotes is intricately regulated and relies on the involvement of multiple protein factors. Transcription factors play crucial roles by binding to specific upstream sequences and thereby ensuring precise regulation of target genes. These factors typically possess a stable DNA-binding domain that interacts with specific DNA fragments, thereby promoting the expression of specific genes. Additionally, most transcription factors contain an activation domain, often composed of disordered regions, which facilitates binding with other protein regulators and plays a significant role in transcriptional regulation. Notably, Richard Young’s group reported that various transcription factors can activate gene expression through phase separation with the Mediator complex [86]. Furthermore, Ding and his research group elucidated that the key transcription factor OCT4 regulates TAD recombination via a phase separation mechanism, promoting somatic cell reprogramming [108]. This study represents the first evidence demonstrating that the phase separation mechanism regulates the higher-order chromatin structure to facilitate cell fate transformation.

Overall, these findings point to an emerging theme that TFs do not simply bind DNA as isolated regulators; rather, they act as dynamic scaffolds that organize local transcriptional environments through phase separation. This concept provides a more integrated explanation for how TF specificity, signaling responsiveness, and chromatin context converge to shape gene-expression programs.

### 5.3. The LLPS in Transcription Cofactors

Transcriptional coactivators, which typically enhance transcriptional activity without directly binding to DNA, often possess intrinsic enzymatic activities that promote gene activation and act as scaffolds for recruiting other chromatin remodeling enzymes [109]. Among these coactivators, MED1 and BRD4 have been extensively studied in the context of LLPS. Sabari et al. provided experimental evidence demonstrating that the transcriptional coactivators BRD4 and MED1 form condensates at super-enhancer (SE) regions. They explained that the MED1 protein incorporates BRD4 and RNAPII into an artificial phase-separated compartment, which could potentially be utilized to “squelch” transcription in nuclear extracts [110]. Based on this therapeutic approach, small-molecule inhibitors of super-enhancer factors such as BRD4 are currently being tested in clinical trials as anticancer therapeutics [111]. Although tumor cells often develop resistance to targeted therapies, combining BRD4 inhibitors with inhibitors of additional transcriptional regulators may help prevent or overcome such resistance [12].

In brief, an important emerging concept is that transcriptional cofactors are not merely passive intermediates between TFs and RNA Pol II. Instead, they actively shape the molecular composition, stability, and functional output of transcriptional condensates, thereby influencing both normal gene regulation and disease-associated transcriptional rewiring.

### 5.4. Transcriptional Condensates in Cancer

Transcriptional condensates have emerged as central organizational units of gene regulation in cancer cells, arising through cooperative interactions among transcription factors, coactivators such as Mediator and BRD4, RNA polymerase II, chromatin-associated proteins, and regulatory RNAs [112]. Rather than functioning as static compartments, they behave as dynamic molecular hubs that integrate signaling inputs and modulate transcriptional burst kinetics. In tumor cells, this framework explains how oncogenic pathways amplify super-enhancer-driven gene expression, maintain lineage identity, and enable rapid adaptive responses to stress or therapy [101]. Despite these advances, several questions remain unresolved: the criteria for distinguishing bona fide liquid–liquid phase separation from alternative clustering remain debated [113]; the extent to which condensates are causal drivers versus correlates of activation is unclear [114]; and how material properties influence target selectivity is poorly understood. Addressing these issues requires rigorous quantitative and perturbation-based approaches. Therapeutic targeting of LLPS is attracting increasing interest, particularly in tumors dependent on aberrant transcriptional condensates. Potential strategies include disrupting multivalent interactions, modulating post-translational modifications that regulate condensate assembly, and targeting critical scaffold components [113,115]. Recent studies on FOXM1 and YAP condensates support feasibility in principle [116]. Nevertheless, clinical translation remains challenging because many phase-separation mechanisms are shared between malignant and normal cells. Future progress depends on identifying tumor-selective condensate dependencies, robust pharmacodynamic biomarkers, and compounds with sufficient specificity.

### 5.5. Therapeutic Strategies Based on LLPS-Mediated Transcriptional Regulation

Enhancers typically span hundreds of base pairs and are cooperatively bound by clusters of multiple transcription factor molecules [117,118]. Transcription factors generally contain two functional parts: a DNA-binding domain (DBD) and one or more independent activation domains (ADs) [119,120]. While the DBD is often well-structured, the ADs of many transcription factors consist of low-complexity amino acid sequences. Evidence indicates that a large number of transcription factors interact with a limited set of coactivator complexes through LLPS condensates. Recent studies have shown that the pluripotency factor OCT4, the estrogen receptor (ER), and the yeast transcription factor GCN4 all undergo phase separation with the Mediator complex, relying on common amino acid motifs or ligands to achieve transcriptional activation and condensate formation [86]. These findings suggest that transcriptional regulation may, at least in part, be driven by the formation of phase-separated condensates.

The core rationale lies in exploiting the “fragility” of pathological condensates. Oncogenic mutations often hijack normal LLPS mechanisms to form hyper-stable pathological condensates that drive transcriptional addiction and genomic instability. These condensates are highly dependent on multivalent interactions, local concentrations, and microenvironmental conditions, creating selective therapeutic windows. Importantly, this approach may spare normal physiological condensates, offering improved specificity [121]. Several breakthrough strategies have been reported. One major direction involves small-molecule modulators that either promote or disrupt condensate formation. For instance, TFAP2β was identified as a key tumor suppressor in esophageal squamous cell carcinoma; the small molecule A6, discovered through virtual screening, enhances TFAP2β phase-separation capacity and suppresses tumor growth in mouse and organoid models [122]. In acute myeloid leukemia, CGX-635, an inhibitor targeting the phase-separation domain of FBL, has demonstrated efficacy in eliminating cancer cells [123].

Another innovative approach is condensate engineering. Researchers have engineered LLPS-enhanced p53 variants (LLPSEp53s) that rewire p53′s transcriptional output—selectively amplifying tumor-suppressor gene programs while weakening pro-tumorigenic neural-associated modules, achieving notable anti-tumor effects in melanoma models [113]. Targeting super-enhancers also shows clinical promise. The small molecule JQ1, which inhibits BRD4, disrupts super-enhancer function and selectively suppresses oncogenes like MYC; BET inhibitors are already in clinical trials [124]. Additionally, in multiple myeloma, compound BBA was found to target the intrinsically disordered region (IDR) of c-Maf, blocking its phase separation and downstream Mtbp/c-Myc oncogenic axis [125].

The Hippo/YAP signaling pathway is a key regulator of cancer cell proliferation, stemness maintenance, and the innate immune response. It has been shown that YAP interacts with multiple transcription factors and functions at super-enhancer regions in the genome [126]. Cai et al. discovered that under hyperosmotic stress conditions, YAP forms phase-separated condensates in both the nucleus and the cytoplasm. These condensates contain distinct protein components and facilitate the redistribution of YAP into the nucleus. Within the nucleus, YAP droplets reorganize genome architecture, thereby promoting sustained expression of YAP target genes [127]. Regarding the transcriptional function of TAZ condensates, Lu et al. found that TAZ condensates colocalize with TEAD4 and other components of phase-separated transcriptional hubs, suggesting a role for TAZ in phase-separated super-enhancers [128]. Further experimentation on the LLPS progress of YAP/TAZ proteins will be necessary to explore the functionality of YAP and TAZ condensates.

### 5.6. Emerging Themes and Future Perspectives

Several emerging themes in LLPS biology are becoming increasingly apparent. First, LLPS should be considered a highly context-dependent process, influenced by protein sequence features, post-translational modifications, RNA interactions, and local physicochemical microenvironmental properties [129,130]. Second, increasing evidence suggests that condensates are functionally heterogeneous, with their material properties closely linked to distinct biological outputs [113]. Third, the interplay between LLPS and genome regulation—including enhancer activity, chromatin architecture, and transcriptional control—has emerged as a major area of interest [112,114]. Fourth, disease-associated phase separation is increasingly recognized not merely as a consequence of molecular dysfunction, but also as a potential driver of pathological progression [129]. Finally, the development of therapeutic strategies targeting condensate dynamics, composition, or physical properties represents a promising frontier, although major challenges remain regarding specificity, delivery, and long-term efficacy [116]. Emerging themes suggest that LLPS is not merely a passive mechanism for concentrating transcriptional components, but a dynamic and context-dependent regulator of enhancer activity, chromatin topology, and transcriptional selectivity. Increasing evidence indicates that the material properties of transcriptional condensates are highly sensitive to post-translational modifications, local RNA concentration, and environmental stress, highlighting their potential roles in both adaptive cellular responses and disease progression.

## 6. Conclusions

LLPS has emerged as a fundamental mechanism underlying a broad spectrum of cellular processes, including cell division, signal transduction, higher-order genome organization, and transcriptional regulation. Despite rapid advances in this field, the mechanisms governing the dynamic assembly, material properties, and physiological functions of biomolecular condensates remain incompletely understood. Increasing evidence indicates that aberrant phase separation, caused by mutations in phase-separating proteins, impaired quality control systems, or altered microenvironmental conditions, contributes to the development of multiple diseases. Therefore, elucidating the principles and consequences of phase transitions will not only deepen our understanding of disease pathogenesis but also provide new opportunities for therapeutic intervention. As this field continues to evolve, research on phase separation is expected to substantially reshape our understanding of cellular organization and biological regulation.

## Figures and Tables

**Table 1:** Representative RNA–protein interactions involved in liquid–liquid phase separation.

RNA	Interaction Protein	Functions	References
miRNA			
miR-7	TNRC6B, AGO2	Promoting irradiation-induced DNA damage repair	[35]
let-7	YTHDF1, AGO2	Promoting P-body formation	[36]
miR-214-3p	Prp (c)	Inhibiting autophagy-related protein 5-dependent autophagy and muscle bundle formation	[37]
miR-233	YBX1	Recruiting miR-233 into exosomes secreted by cultured cells	[38]
circRNA			
circASH2	Y-box binding protein 1 (YBX1)	Augmenting TPM4 transcript decay	[39]
circVAMP3	CAPRIN1	Promoting stress granule formation and downregulating Myc protein levels	[40]
ciRS-7	TNRC6B, AGO2	Promoting irradiation-induced DNA damage repair	[35]
lncRNA			
SNHG9	LATS1	Inhibiting LATS1-mediated YAP phosphorylation	[41]
NEAT1	TDP-43	Excessive cytoplasmic translocation of TDP-43 to form stress granules	[42]
MALR	ILF3	Activation of HIF1α signaling to promote cancer progression	[43]
NORAD	Pumilio	Formation of phase-separated PUM condensates	[44]
GATA2-AS1	FUBP3	Decrease in SUZ12 activity and epigenetic upregulation of GATA2	[45]
LncFASA	PRDX1	Disrupting intracellular ROS homeostasis	[46]
GIRGL	CAPRIN1	Inducing stress granule formation	[47]

## Data Availability

No data was used for the research described in the article.

## Abbreviations

AGO2: Argonaute 2; TNRC6B: Trinucleotide repeat containing 6B; YTHDF1/YTHFD1: YTH N6-methyladenosine RNA binding protein 1; YBX1: Y-box binding protein 1; circRNA: circular RNA; PrP©: cellular prion protein.
